# Assessment of Sport Nutrition Knowledge, Dietary Practices, and Sources of Nutrition Information in NCAA Division III Collegiate Athletes

**DOI:** 10.3390/nu13092962

**Published:** 2021-08-26

**Authors:** Dylan J. Klein, Kaitlyn M. Eck, Alan J. Walker, Joseph K. Pellegrino, Daniel J. Freidenreich

**Affiliations:** 1 Department of Health and Exercise Science, Rowan University, Glassboro, NJ 08028, USA; 2 Department of Nutrition and Dietetics, Marywood University, Scranton, PA 18509, USA; keck@maryu.marywood.edu; 3 Department of Exercise Science, Lebanon Valley College, Annville, PA 17003, USA; alwalker@lvc.edu; 4 Department of Health and Human Performance, University of Scranton, Scranton, PA 186510, USA; joseph.pellegrino@scranton.edu; 5 Department of Exercise and Sport Science, University of Wisconsin-La Crosse, La Crosse, WI 54601, USA; dfreidenreich@uwlax.edu

**Keywords:** sport nutrition knowledge, dietary practices, collegiate athletes, sources of nutrition information

## Abstract

Nutrition knowledge is a critical component of meeting sport nutrition guidelines. The present study aimed to evaluate the sport nutrition knowledge of National Collegiate Athletic Association (NCAA) Division III (DIII) athletes using a validated questionnaire, and to assess the dietary practices and sources of nutrition information in this population. A total of 331 student-athletes (*n* = 149 males, *n* = 181 females, *n* = 1 no sex indicated) completed the questionnaire. The mean score for total sport nutrition knowledge was 6.49 ± 8.9 (range −49 to 49) with a mean percent (%) correct score of 36.9 ± 19.1%. Athletes who had a previous college-level nutrition course (*n* = 62) had significantly higher (*p* < 0.05) total sport nutrition, carbohydrate, and hydration knowledge compared to those who did not (*n* = 268). Individual sport athletes (*n* = 90) scored significantly higher (*p* < 0.05) on hydration and micronutrients knowledge than team sport athletes (*n* = 237), while females scored higher than males for hydration knowledge (*p* < 0.05). The majority of athletes reported sensible dietary habits, such as not frequently skipping meals and eating carbohydrate and protein foods peri-workout. Athletes also reported their primary sources of nutrition information, the top three sources being social media, coaches, and athletic trainers, despite most frequently rating registered dietitians/nutritionists as “extremely knowledgeable”. Despite low sport nutrition knowledge, NCAA DIII collegiate athletes practiced seemingly prudent dietary habits but lacked exposure to high-quality sources of nutrition information.

## 1. Introduction

A well-chosen diet plan is critical for optimizing sport performance and the promotion of beneficial training adaptations [1]. Compared to a non-athletic population, athletes require greater amounts of energy, fluids, and macronutrients (i.e., carbohydrate, protein and fat) in order to sustain vigorous training and recovery, and to support physiological functions outside of the energy demands of sport [1,2]. Prolonged failure to meet these demands places an athlete at risk of developing a condition known currently as relative energy deficiency in sport (RED-S) [3]. This condition is defined by the intersection of low energy intake normalized to fat-free mass (i.e., low energy availability [LEA]), poor bone mineralization, and impaired reproductive function that, together, can impair performance and health [4,5]. Unfortunately, athletes commonly fail to meet recognized sport nutrition guidelines for energy, fluid, and macronutrients [6,7,8], placing them at risk for LEA and RED-S [5]. Understanding the development and prevention of poor dietary intakes and the accompanying conditions of LEA and RED-S has become the focus of much research in recent years [9,10]. While the causative factors for failing to meet recognized sport nutrition guidelines are likely multifactorial (e.g., taste, convenience, and access to resources [11]), one postulated reason for the mismatch between the requisite energy demands of sport and actual dietary intakes in athletes is poor sport nutrition knowledge.

Sport nutrition knowledge can be defined as the understanding of the nutrition-related factors that can affect training, athletic performance, and recovery from sport. This knowledge goes beyond general nutrition knowledge that may only focus on food groups, sources of nutrients, and overall general health requirements that fail to acknowledge the special needs of high-performing athletes. Having a sound understanding of sport nutrition can allow the athlete to best complement the demands of their training and optimally promote performance and recovery. Research suggests that higher nutrition knowledge is correlated with better diet quality [12,13] and interventions aimed at improving nutrition knowledge in athletes leads to better dietary intakes [14,15]. Together, this suggests that knowledge is a critical component in addressing optimal nutrition in athletes.

In recent years, a number of studies have been conducted assessing the level of nutrition knowledge in a wide range of athletes [16,17,18]. While some studies suggest that athletes may have sufficient nutrition knowledge [19,20,21], others suggest that the nutrition knowledge is inadequate [18,22,23,24,25], particularly in collegiate athletes [16,18,23]. In this population, National Collegiate Athletic Association (NCAA) Division I (DI) athletes have been the primary subjects of interest, with little attention being paid to Division IIII (DIII) athletes that constitute the largest proportion of participating NCAA institutions (approximately 40% of total athletes). While DI athletes have been shown to exhibit poor nutrition knowledge [16,17,18], many DI institutions have vast resources dedicated to some form of nutritional support (e.g., dietitians, training tables, and yearly stipends that offset living costs). This, it can be argued, may minimize the potentially negative impacts of poor nutrition knowledge, given that access to dietitians and prudent nutritional choices are more readily available to these athletes. To the contrary, DIII institutions have very little resources devoted to their athletes, thus placing greater demands on the athletes to seek out useful information and to make well-informed decisions about nutrition. As such, nutrition knowledge is likely to be critical in these athletes who lack adequate resources and may be exposed to a variety of conflicting and potentially ill-informed sources of information.

To date, very few studies have exclusively assessed the sport nutrition knowledge of DIII collegiate athletes, their dietary habits, and sources of nutrition information. In order to address this knowledge gap, the present study aimed to evaluate the sport nutrition knowledge of NCAA DIII male and female collegiate athletes using an appropriately validated sport nutrition knowledge questionnaire. We also aimed to assess the dietary practices and sources of nutrition information in this population.

## 2. Materials and Methods

### 2.1. Subjects

The Rowan University Institutional Review Board (IRB) approved this study, and all student-athletes voluntarily agreed to participate prior to taking the digitally provided, anonymous, online survey. Subjects had the right to exit the survey at any time. All participants (*n* = 149 males, *n* = 181 females; *n* = 1 no sex indicated) were 18 years or older and were recruited from five NCAA DIII universities that span the Mid-Atlantic and Midwest regions. Complete subject demographic information is located in Table 1. To include as many participants as possible, a recruitment letter was used and each investigator cooperated with their respective athletics department director(s) and/or directly with coaches/teams to encourage their athletes to participate (approximately 2497 potential student-athlete respondents). In some instances, encouragement to participate was spread via word of mouth. Student-athletes did not receive incentives for participation. Recruitment took place between October and December 2020.

### 2.2. Assessment of Sport Nutrition Knowledge and Dietary Habits

The dietary habits and sport nutrition knowledge survey that participants completed was 85 questions in length. The sport nutrition knowledge component consisted of a validated, 49-item questionnaire [26], broken into six sections (carbohydrate (11 questions), protein (9 questions), fat (7 questions), hydration (7 questions), micronutrients (7 questions), and weight management (8 questions)), all of which were explicitly related to sport nutrition. In addition to this assessment, the athletes also completed an additional 36 questions pertaining to demographic information (11 questions), dietary habits (18 questions), and sources of nutrition information (7 questions). The dietary habits and sources of nutrition information questions were not validated. The survey was administered online via Qualtrics software (Provo, UT, USA) and taken at the athletes’ leisure.

The 49-item sport nutrition knowledge instrument used in this study was developed by Karpinksi et al. [26]. This instrument (Cronbach α 0.843; test-retest *r* = 0.951, *p* < 0.001) was validated in adult athletes, a majority (61.9%) of whom were collegiate athletes spanning NCAA DI, II, and III schools. Additionally, the questionnaire was developed based on the most recent 2016 Joint Position Statement from the American College of Sports Medicine (ACSM), the Academy of Nutrition and Dietetics (AND), and Dietitians of Canada (DC) on Nutrition and Athletic Performance [1] as well as other published sport nutrition recommendations and standards [27,28]. Given these factors, we believed it to be the most appropriate questionnaire at the time of assessment.

### 2.3. Scoring

Scoring of the sport nutrition knowledge instrument was done as outlined by Karpinski et al. [26]. Participants received +1 point for each correct answer, −1 point for each incorrect answer, and +0 points for “Don’t know”. In instances where more than one response was indicated, the response was coded as “Don’t know” and a score of +0 was given. As such, the possible range of scores ranged from −49 to 49. Individual section scores (i.e., carbohydrate, protein, fat, hydration, micronutrients, and weight management) were also calculated within each of the six sections. Additionally, to obtain how many questions were answered correctly, a “% correct” score was calculated by dividing the total number of correct responses by the total number of questions. This was done for the entire survey as well as each individual section.

### 2.4. Statistical Analyses

Participant characteristics, dietary habits, and sources of nutrition knowledge are presented using descriptive statistics. All statistical analyses were carried out using SPSS software version 28.0 (IBM Corporation, Chicago, IL, USA). Student’s *t*-test was used to compare differences in nutrition knowledge scores between sexes, those having had a college-level nutrition course or not, and between individual and team sport athletes. An individual sport athlete was anyone who reported competing in the following sports: golf, track and field, cross country, swimming and diving, gymnastics, and tennis. All other reported sports were considered team sports (i.e., baseball, softball, basketball, ice hockey, field hockey, soccer, volleyball, lacrosse, and football). Significance was set at alpha < 0.05. Knowledge data are presented as means ± standard deviations (SD).

## 3. Results

### 3.1. Participants

There were approximately 2497 student-athletes that were contacted as part of the study. A total of 489 athletes initiated the survey (19.5%), and 331 ultimately completed it (13.2%). Athletes’ physical characteristics are located in Table 1. Athletes from 10 different sports were represented by the male respondents, and a total of 12 different sports were represented by the female respondents. A full breakdown of the sports represented, by sex, can be located in the Supplementary Materials (Table S1). Of the 331 total respondents, 42 individuals had previously been counseled by a dietitian or nutritionist (66.7% female) and 62 individuals had taken a college-level nutrition course (66.1% female). Of the participants who reported their declared major (*n* = 331), only four respondents declared nutrition and/or dietetics as their college major (100% female). A total of 57 female and 33 male respondents declared a major in another health- or exercise-related field (e.g., exercise science, athletic training, nursing, physical therapy, and kinesiology).

### 3.2. Total Sample Analyses

Sport nutrition knowledge scores for the entire 49-item survey ranged from −38 to 49, with the mean score being 6.4 ± 8.9. Ranges and mean scores for the individual sections were also calculated, and a detailed summary is located in Table 2.

In order to determine the number of questions answered correctly without the negative influence of incorrect responses, we also calculated the correct score percentage for the entire survey. For the entire sample, this amounted to a mean score of 36.9 ± 19.1%.

### 3.3. Sex Differences

Differences between scores by sex are located in Table 2. There were no significant sex differences (*p* > 0.05) between males and females for total sport nutrition knowledge, carbohydrate, protein, fat, or weight management categories. There was, however, a significant difference (*p* = 0.016) between males and females for hydration knowledge, with females scoring higher than males (1.4 ± 1.8 vs. 0.9 ± 2.0, respectively). There was no significant sex difference for the percentage of correct scores for the entire survey (35.8 ± 20% for males vs. 37.8 ± 18.4% for females; *p* = 0.340) or for any individual section (*p* > 0.05).

### 3.4. Previous Nutrition Course Differences

Differences in sport nutrition knowledge between athletes who had taken a college-level nutrition course and those who had not are located in Table 2. There were no significant differences (*p* > 0.05) in scores between those who had a nutrition course and those who did not for total sport nutrition knowledge, fat, hydration, micronutrients, and weight management categories. There were, however, significant differences for carbohydrate (*p* = 0.035) and protein (*p* = 0.025) knowledge scores, with those having taken a nutrition course showing a higher and lower score for carbohydrate and protein knowledge, respectively. When analyzed by “% correct”, those who had a previous college-level nutrition course had statistically significantly higher scores for total nutrition knowledge (41.8 ± 18.8% vs. 35.8 ± 19.1%, *p* = 0.024), carbohydrate knowledge (51.9 ± 21.6% vs. 40.4 ± 20.2%, *p* = 0.0001), and hydration knowledge (42.9 ± 26.1% vs. 35.3 ± 25.7%, *p* = 0.034).

### 3.5. Team vs. Individual Sport Differences

Differences in sport nutrition knowledge between team and individual sport athletes are located in Table 2. The only significant difference (*p* = 0.037) in scores between the groups was for hydration knowledge, with individual sport athletes scoring higher than team sport athletes (1.5 ± 2.1 vs. 1.0 ± 1.8, respectively). There were no significant differences (*p* > 0.05) between the groups for total sport nutrition knowledge, carbohydrate, protein, fat, micronutrient, and weight management categories. There were significant differences for the percentage of correct scores between individual and team sport athletes for hydration knowledge (41.9 ± 26% vs. 34.4 ± 25.4%, *p* = 0.019) and micronutrient knowledge (40.1 ± 27.1% vs. 31.4 ± 24.6%, *p* = 0.006).

### 3.6. General Dietary Habits

As part of the survey, we also asked athletes about their general dietary habits. Less than half of male (45.6%) and female (41.0%) athletes reported eating breakfast every day. Male athletes were more likely to eat their lunch and dinner meals in the cafeteria, whereas female athletes generally had an even distribution between self-prepared meals and meals from the cafeteria (Table 3). The majority of male and female athletes reported eating three meals per day (61.7% vs. 60.7%, respectively), whereas a higher percentage of male athletes reported eating greater than four meals per day compared to female athletes (14.8% vs. 2.2%, respectively; Table 3). The majority of both sexes reported not frequently skipping meals (78.5% males, 65.2% females; Table 3) and for those that did skip meals, the highest reported reason for skipping meals was schedule/time constraints (31.5% males vs. 28.9% females; Table 3). Snacking frequency between the sexes did not exhibit many differences; however, male athletes reported lower consumption of fruit and vegetable snacks and higher consumption of protein bars/shakes compared to female athletes (12.1% vs. 24.9% and 33.6% vs. 13.8%, respectively; Table 3).

### 3.7. Sport-Related Dietary Habits

**Figure 1:**
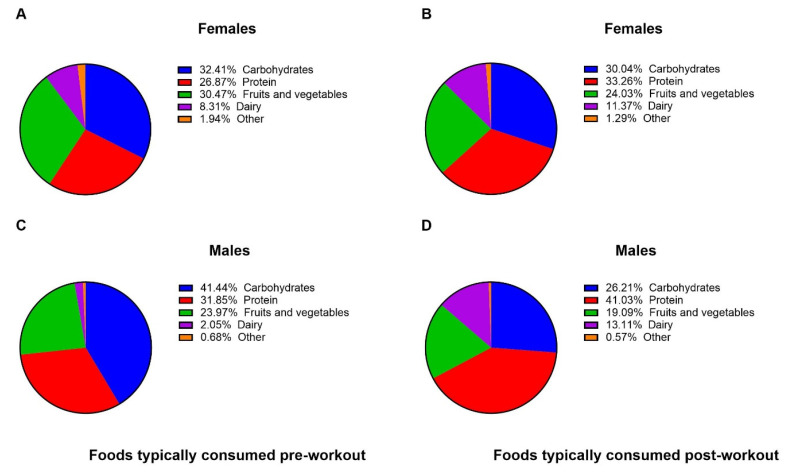
Pre- and post-workout food choices of athletes. Athletes were asked to select all foods they typically consume before and following workouts/practices. Data are presented as percentage of all responses cast among all options (listed in the figure). (**A**): *n* = 361 total responses; (**B**): *n* = 466 total responses; (**C**): *n* = 292 total responses; (**D**): *n* = 351 total responses.

With regard to sport-related dietary habits, the majority of both sexes reported drinking water during workouts and/or practices (95.3% males and 96.7% females; Table 4). Similarly, the majority of both sexes reported eating within one hour of workouts and/or practices (67.1% males vs. 58.9% females) and within one-to-two hours post-workouts and/or practices (49.7% males vs. 59.4% females; Table 4). When asked to select which food types they typically consume before and after workouts/practices, both sexes reported consuming mostly carbohydrate, protein, and fruit and vegetable foods. A detailed summary of the foods typically consumed before and after workouts/practices can be found in Figure 1.

### 3.8. Motivations for Dietary Habits and Supplement Use

A detailed summary of the motivations for dietary habits and supplement use can be found in Table 5. A breakdown of the types of supplements typically consumed by the athletes can be found in Supplementary Figure S1. The supplement categories that received the most responses from females were a multi-vitamin/mineral supplement (37.7%), protein supplements (25.3%), and pre-workout with caffeine (9.3%). The supplement categories that received the most responses from males were protein supplements (31.4%), followed by “I do not take supplements regularly” (16.4%) and multi-vitamin/mineral (14.3%).

### 3.9. Sources of Nutrition Information

Subjects were asked to rank order their top three sources of nutrition information (*n* = 925 total responses). The category that most athletes ranked in their top three sources of nutrition information was Social Media (20.2%), followed by Coaches (16.8%), Athletic Trainers (15.5%), Physician (12.9%), Registered Dietitian/Nutritionist (8.5%), Professor (6.6%), Other (5.9%), Academic Journals (5.5%), Not Applicable—I have never sought out nutrition information (4.9%), and Popular Magazines (3.2%). When asked to rank order the top three persons they “feel most comfortable discussing their nutrition needs with” (*n* = 910 total responses), athletes chose Athletic Trainer (30%), followed by Coach (22.2%), Registered Dietitian/Nutritionist (22.2%), Physician (20.3%), Other (4.9%), Professor (4.3%), and Not Applicable—I have never sought nutrition information (3.1%).

Athletes were asked to rate the adequacy of nutrition knowledge of Coaches, Athletic Trainers, Registered Dietitians/Nutritionists, and Physicians. Of the professionals, Registered Dietitians/Nutritionists received the highest adequacy ratings, followed by Physicians, Athletic Trainers, and Coaches (Table 6).

## 4. Discussion

To the best of the authors’ knowledge, this is the largest nutritional knowledge assessment of NCAA DIII student-athletes using an appropriate, validated, sport nutrition knowledge questionnaire. We found that male and female NCAA DIII student-athletes, from multiple institutions, spanning a variety of sporting disciplines exemplified low sport nutrition knowledge, placing them at risk for poor dietary intakes that could negatively affect training, recovery, and performance. This was shown in the mean score for the total survey being 6.39 (range of −38 to 49) and a correct score percentage between approximately 35% and 40% regardless of sex, having had taken college-level nutrition course, or participation in a team or an individual sporting discipline. While it is difficult to compare across similar studies due to the high heterogeneity in assessment tools and athlete populations, our study showed a percentage of correct scores below the typical 45–65% range reported in the literature [16,17,18]. In a direct comparison to the only other study that utilized this assessment tool, the developers of the survey found a mean score of 15.9 and a correct score percentage of 55.4% in their population of adult athletes, a majority of whom (61.9%) were collegiate athletes and female (70.8%) [26]. Regardless, based on the previously reported cutoff of 75% for exhibiting “adequate” or “excellent” knowledge [22,25,29], our results are in line with the majority of studies showing inadequate nutrition knowledge in collegiate athletes [16]. Furthermore, these results indicate that NCAA DIII collegiate athletes, who typically lack robust institutional resources, are particularly in need of sport nutrition education and could benefit from greater institutional support.

Previous studies have shown nutrition knowledge differences between the sexes, with females scoring higher than males when differences are present [18,30]. This finding, however, is not universal across studies, with the majority showing no differences [16]. Our findings are in agreement with the majority of research showing no sex differences in total sport nutrition knowledge; however, we did uncover a difference in hydration knowledge between males and females when using the scoring instructions outlined by Karpinski et al. [26] (Table 2). To the best of our knowledge, while no studies have shown higher hydration knowledge of female athletes compared to male athletes, one study by Volpe et al. [31] did show that a greater percentage of male collegiate athletes were hypohydrated compared to female athletes (47% vs. 28%, respectively), potentially indicating better knowledge and hydration practices in females.

Unsurprisingly, athletes who had taken a college-level nutrition course had significantly higher (% correct) knowledge scores than those who had not taken a nutrition course. These findings are in agreement with previous studies showing greater nutrition knowledge in athletes who had previously taken a nutrition course compared to those that did not [32,33]. While a college-level nutrition course obviously imparted greater knowledge, it was still not effective at promoting adequately high sport nutrition knowledge, as these athletes did not score above 42% on the instrument. This highlights the specialized information that athletes require regarding nutrition and performance and the need for greater resources to address their specific nutritional needs.

Interestingly, individual sport athletes scored higher (% correct) than team sport athletes for both hydration and micronutrients knowledge. This is in partial agreement with findings by Weeden et al. [34], who found that individual sport athletes had higher nutrition knowledge than team sport athletes. A possible explanation may be due to differences in “self-regulatory” behaviors between these athlete types. Indeed, Jonker et al. [35] demonstrated that athletes participating in individual sport disciplines reported higher ratings on “planning” and “effort” skills than team sport athletes. Regardless, given the low knowledge scores across all groups, the fact remains that collegiate athletes are greatly in need of sport nutrition education that could help them optimize training, recovery, and performance.

One outcome of poor nutrition knowledge is poor dietary practices that fail to support training needs. Despite exhibiting inadequate sport nutrition knowledge, our sample reported relatively prudent dietary practices, such as not frequently skipping meals, eating at least three meals per day, and consuming multiple snacks per day. Interestingly, however, just less than half of males and females reported eating breakfast every day, potentially highlighting a barrier to meeting total energy needs throughout the week in some athletes. Additionally, males were less likely to report choosing fruit and vegetable snacks compared to females, whereas the reverse was true for protein bars/powders. This, in part, reflects the athletes’ reported motivations for their dietary and supplementation practices. Indeed, males reported “performance” and “recovery” at higher rates whereas females reported “general health” more frequently.

Sport-related dietary habits were also a part of the nutrition assessment in the present study. Despite low sport nutrition knowledge, athletes reported eating within one hour pre- and within one to two hours post-workouts/practices, selecting mostly carbohydrate and protein-based foods for these meals. This complies with recognized sport nutrition guidelines for macronutrient selection and timing of nutrients to promote performance and recovery [1,2]. However, despite these practices, it is unclear the extent to which these athletes actually meet the energy and relative carbohydrate and protein needs for their given level of training. Indeed, the relevant literature has consistently shown that the dietary intakes of collegiate athletes do not typically meet the recommended nutrient intakes [6,7,36,37,38]. This could potentially be due to a mismatch between perceptions of dietary requirements and actual needs [25]. Thus, while dietary habits may be prudent, gross intakes may not be sufficient, placing athletes at risk for LEA and developing RED-S [10] and overtraining syndrome [39].

The vast majority (>95%) of athletes of both sexes also reported drinking water during workouts/practices. While water is a logical choice for hydration purposes, athletes who engage in vigorous activity for longer than 60 min may need additional nutrients, such as carbohydrate and electrolytes, to counter high sweat rates, promote performance, and delay fatigue [40]. Thus, while athletes acknowledged, in practice, hydration as a key component to optimal sport nutrition, the present data highlight the potential of specific hydration strategies as a key aspect for improving sport nutrition knowledge in athletes.

Given that NCAA DIII collegiate athletes typically do not have access to a sports dietitian or the level of nutrition resources provided to DI athletes at larger institutions, we also addressed sources of nutrition knowledge in our sample, as this is likely to play a large role in these athletes’ decisions. The category ranked most frequently as the number one, two, or three source of nutrition knowledge was social media, followed by coaches and athletic trainers. This was reflected in the athletes’ reporting of who they feel most comfortable discussing their nutritional needs with, with athletic trainers and coaches ranking the highest. Torres-McGehee at al. [22] similarly showed coaches and athletic trainers as being primary resources for nutrition knowledge in collegiate athletes. This is likely due to the high degree of trust and high frequency with which the athletes interact with these individuals. Furthermore, two more recent studies, one by Kimmel et al. [41] and another by Trakman et al. [42], also showed the internet as being one of the primary sources of nutrition information for athletes. While there is certainly a distinction between the “internet” and “social media” (as we presented it), the present study coupled with the recent findings by Kimmel and Trakman highlight the ever-growing influence of online digital media as a primary source of nutrition information for athletes. It further stresses the potential need for health professionals and coaches to counter low-quality nutrition information. Given that the athletes in the present study reported dietitians as the most knowledgeable with regard to nutrition information, this further emphasizes the importance of having not only sport nutritionists/dietitians in athletic settings, but having competent professionals whom the athletes trust for their information.

One strength of the present study includes the use of an appropriate, validated sport nutrition knowledge instrument that is based, in part, on the most recent sport nutrition guidelines set forth by the ACSM, AND, and DC [1] as well as additional published sport nutrition recommendations and standards [27,28]. Many previous studies suffer from not using validated surveys, using questionnaires that were validated in populations that are not indicative of collegiate athletes (e.g., adolescents) [16,43], or using (now) outdated information regarding sport nutrition guidelines [22,29]. While this study did not assess general nutrition knowledge, the instrument used applies explicitly to sport-specific nutrition information most relevant to athletes. Given the rigorous validation process of the current instrument by Karpinski et al. [26], we believe this study adds much-needed and valuable information on the sport nutrition knowledge proficiency of collegiate athletes. Another strength of this study is the large sample of collegiate athletes (*n* = 331), of both sexes, from various similar institutions, that span a wide variety of sporting disciplines.

This study is not without its limitations. For one, we did not assess actual dietary intakes as aforementioned, nor did we use a validated set of questions related to dietary habits and sources of nutrition information. As such, the qualitative data in the present study should be interpreted with some caution. Furthermore, we also failed to assess ethnicity or socioeconomic status, two factors that are likely to play crucial roles in nutrition knowledge and dietary behaviors [44]. For example, in a recent study by Jagim et al. that assessed NCAA DIII collegiate athletes [25], one of the reported reasons for skipping meals was financial restrictions, particularly among the female respondents. Thus, future studies should aim to include additional factors and barriers to nutrition that can better help contextualize the data and tease out the most important contributors to dietary practices and knowledge in athletes.

Another limitation of the study was the length of the survey. In total, our survey encompassed 85 questions related to demographics, dietary habits, sources of nutrition information, and sport nutrition knowledge. While 331 student-athletes completed the questionnaire with a fairly even distribution of male and female respondents, approximately 2497 students were contacted, while 489 initially opened and agreed to take the survey and 158 did not complete it (13.2% response rate). As such, this likely contributed to bias in the sample. Finally, our results are likely only generalizable to athletes with similar educational backgrounds from similar collegiate institutions (i.e., DI, DII, and DIII).

## 5. Conclusions

Sport nutrition knowledge is a critical component for optimizing dietary habits that promote beneficial training adaptations in athletes. In the present study, NCAA DIII collegiate athletes exhibited poor sport nutrition knowledge, regardless of sex, having had a college-level nutrition course or not, and whether they participated in an individual or team sport discipline. Despite the lack of formal knowledge, most athletes reported seemingly prudent dietary practices when it came to skipping meals and pre- and post-workout/practice nutrition. Furthermore, while we did observe differences in knowledge between groups of athletes (i.e., by sex, having had a nutrition course, and sport type) that agree with previous research findings, the overall low scores across all groups point toward a need for greater nutrition education in collegiate athletes, even in those who have taken a college-level course in nutrition. Given that most athletes in the present study relied upon social media and those most proximal to them for nutrition information (i.e., coaches and athletic trainers who are not technically experts in sport nutrition knowledge), it is imperative that more institutional support is provided to these types of athletes. This should include efforts that enhance sport nutrition knowledge, identify barriers to poor nutrition, and employ registered sports dietitians who can deliver the most reliable dietary information.

## Figures and Tables

**Table 1:** Athlete characteristics.

Characteristic	Males (*n* = 149)	Females (*n* = 181)
Age (yrs.)	19.6 ± 1.6	19.4 ± 1.2
Height (cm)	181.0 ± 7.6	166.7 ± 7.6
Body mass (kg)	82.9 ± 13.6	64.2 ± 9.8
Body mass index (kg/m^2^)	25.2 ± 3.8	23.1 ± 2.9

Data presented as means ± SD.

**Table 2:** Athlete sport nutrition knowledge scores.

		Total Sample (*n* = 331)		Sex ^†^		Nutrition Course ^††^		Sport Type ^†††^	
	No. of Items	Range (Min, Max)	Mean ± SD	Males (*n* = 149)	Females (*n* = 181)	Yes (*n* = 62)	No (*n* = 268)	Team (*n* = 237)	Individual (*n* = 90)
Total survey	49	−38, 49	6.4 ± 8.9	5.7 ± 9.9	7.0 ± 8.0	5.9 ± 9.1	6.5 ± 8.8	6.1 ± 8.8	6.9 ± 9.2
Carbohydrate	11	−11, 11	2.5 ± 2.7	2.5 ± 2.9	2.5 ± 2.5	3.1 ± 3.2	2.3 ± 2.6 ^b^	2.5 ± 2.7	2.4 ± 2.7
Protein	9	−9, 9	−0.8 ± 2.9	−0.9 ± 3.1	−0.7 ± 2.6	−1.5 ± 3.2	−0.6 ± 2.7 ^b^	−0.8 ± 2.8	−0.7 ± 3.1
Fat	7	−5, 7	0.8 ± 1.8	0.7 ± 1.9	0.9 ± 1.8	0.7 ± 1.9	0.9 ± 1.8	0.8 ± 1.8	0.8 ± 2.0
Hydration	7	−5, 7	1.2 ± 1.9	0.9 ± 2.0	1.4 ± 1.8 ^a^	1.2 ± 2.1	1.2 ± 1.9	1.0 ± 1.8	1.5 ± 2.1 ^c^
Micronutrients	7	−5, 7	0.9 ± 2.0	0.7 ± 2.1	1.3 ± 1.9	0.7 ± 2.1	1.0 ± 2.0	0.8 ± 1.9	1.1 ± 2.3
Weight management	8	−6, 8	1.7 ± 2.5	1.8 ± 2.4	1.7 ± 2.4	1.6 ± 2.2	1.8 ± 2.4	1.8 ± 2.5	1.7 ± 2.2

Data are presented as means ± SD. Scoring based on instructions outlined by Karpinski et al. [26]; +1 for correct, −1 for incorrect, +0 for “Don’t know”; score range: −49 to 49. ^†^ *n* = 330, one athlete did not indicate their sex. ^††^ *n* = 330, one athlete did not indicate whether they had taken a nutrition course or not. ^†††^ *n* = 327, four athletes participated in both sports types. ^a^ *p* < 0.02, significantly different than males based on Student’s *t*-test. ^b^ *p* < 0.05, significantly different than athletes who have taken a nutrition course based on Student’s *t*-test. ^c^ *p* < 0.05, significantly different than team sport athletes based on Student’s *t*-test.

**Table 3:** General dietary habits of athletes.

Category	Males	Females
	(*n* = 149)	(*n* = 181)
How often do you typically eat breakfast?		
Every day	45.6%	41.4%
3–5 days/week	26.8%	34.3%
1–2 days/week	10.1%	12.7%
I typically don’t eat breakfast	17.4%	11.6%
What type of lunch do you eat most days?		
Self-prepared	33.6%	49.2%
Cafeteria	63.1%	42.5%
Take-out or fast-food	2.7%	2.2%
I typically don’t eat lunch	0.7%	6.1%
What type of dinner do you eat most days?		
Self-prepared	36.2%	53.0%
Cafeteria	59.7%	44.8%
Take-out or fast-food	3.4%	2.2%
I typically don’t eat dinner	0.7%	—
Typical number of meals per day:		
4+ per day	14.8%	1.7%
3 per day	61.7%	61.3%
2 per day	23.5%	35.4%
1 per day	—	1.7%
Typical number of snacks per day:		
4+ per day	10.7%	8.8%
3 per day	26.2%	22.1%
2 per day	38.9%	46.4%
1 per day	20.1%	18.2%
I don’t snack	4.0%	4.4%
What types of snacks do you typically eat?		
Cakes, sweets, pastries	3.4%	2.8%
Chips, popcorn, pretzels, crackers	28.9%	35.4%
Fruit or vegetables	12.1%	24.9%
Protein bars/shakes	33.6%	13.8%
Yogurt, cheese, dairy	5.4%	7.7%
Cereal/granola bars	16.8%	15.5%
Do you frequently skip meals?		
Yes	21.5%	34.8%
No	78.5%	65.2%
If you frequently skip meals, which option best describes why? *		
Time schedule/constraints	31.5%	28.9%
Not hungry	9.1%	22.5%
Limited access	4.9%	1.7%
Inability to prepare meals	3.5%	0.6%
I almost never skip meals	51.0%	46.2%

Data are presented as percentage of responses (%). * *n* = 143 males, *n* = 173 females.

**Table 4:** Sport-related dietary habits of athletes.

Category	Males	Females
	(*n* = 149)	(*n* = 181)
Which fluid do you typically drink during workouts/practices? *		
Water	95.3%	96.7%
Soft drinks	—	—
Fruit juice	—	—
Sports drinks	4.1%	2.2%
Other low-calorie or zero-calorie	—	1.1%
beverages		
I don’t drink fluids during workouts/practices	—	—
How soon before workouts/practices do you typically eat? **		
Within 1 h	67.1%	58.9%
Between 1–2 h before	30.9%	39.4%
Between 2–3 h before	2.0%	1.1%
More than 3 h before	—	0.6%
How soon after workouts/practices do you typically eat? **		
Within 1 h	8.1%	9.4%
Between 1–2 h after	49.7%	59.4%
Between 2–3 h after	38.3%	27.2%
More than 3 h after	4.0%	3.9%

Data are presented as percentage of responses (%). * *n* = 148 males. ** *n* = 180 females.

**Table 5:** Motivations for dietary habits and supplementation use.

Category	Males	Females
	(*n* = 149)	(*n* = 181)
How often do you take dietary supplements? *		
Every day	29.5%	28.9%
3–5 times per week	34.5%	27.5%
1–2 times per week	8.6%	6.3%
<1 time per week	27.3%	37.3%
Pick the best reason(s) for taking dietary supplements. (Check all that apply.):		
Performance	19.8%	10.8%
Recovery	22.4%	13.8%
Body composition/physique	16.9%	6.9%
Focus/energy	12.0%	10.0%
General health	17.8%	26.5%
I do not take any supplements	11.1%	31.9%
What are the primary reason(s) for your eating habits? (Check all that apply.):		
Performance	24.4%	19.5%
Recovery	19.2%	13.7%
Body composition/physique	18.7%	19.7%
Focus/energy	14.3%	17.0%
General health	22.3%	26.2%
I do not consider my eating habits all that much	1.1%	4.0%

Data are presented as percentage of responses (%). * *n* = 142 females.

**Table 6:** Athletes’ perception of nutrition knowledge of coaches, athletic trainers, dietitians/nutritionists, and physicians.

Category	Males	Females
	(*n* = 149)	(*n* = 181)
In your experience, how would you rate the adequacy of nutrition knowledge of coaches? *		
Extremely knowledgeable	8.7%	6.1%
Very knowledgeable	30.9%	36.1%
Moderately knowledgeable	38.9%	37.8%
Slightly knowledgeable	14.8%	15.6%
Not knowledgeable at all	6.7%	4.4%
In your experience, how would you rate the adequacy of nutrition knowledge of athletic trainers?		
Extremely knowledgeable	20.1%	17.1%
Very knowledgeable	51.7%	47.5%
Moderately knowledgeable	24.8%	30.4%
Slightly knowledgeable	2.7%	3.9%
Not knowledgeable at all	0.7%	1.1%
In your experience, how would you rate the adequacy of nutrition knowledge of dietitians/nutritionists?		
Extremely knowledgeable	71.1%	77.0%
Very knowledgeable	22.8%	18.6%
Moderately knowledgeable	4.7%	3.3%
Slightly knowledgeable	1.3%	0.5%
Not knowledgeable at all	—	0.5%
In your experience, how would you rate the adequacy of nutrition knowledge of physicians?		
Extremely knowledgeable	30.2%	39.3%
Very knowledgeable	49.7%	45.4%
Moderately knowledgeable	18.1%	14.2%
Slightly knowledgeable	2.0%	0.5%
Not knowledgeable at all	—	0.5%

Data are presented as percentage of responses (%). * *n* = 180 females.

## Data Availability

De-identified data can be made available upon request.

## Supplementary Materials

The following are available online at https://www.mdpi.com/article/10.3390/nu13092962/s1, Table S1: Breakdown of reported sports disciplines by sex; Figure S1: Supplements typically consumed by athletes.
