# Supplementary material for: Assessment of Sport Nutrition Knowledge, Dietary Practices, and Sources of Nutrition Information in NCAA Division III Collegiate Athletes
Source: Nutrients. 2021 Aug 26;13(9):2962. doi: 10.3390/nu13092962 (PMC8470142; doi:10.3390/nu13092962)
Supplement: Supplementary file 1 [file nutrients-13-02962-s001.zip › nutrients-1327733-supplementary.pdf]

Supplementary Materials

Tables

Supplementary Table S1. Breakdown of reported sports disciplines by sex.

**Sport**	**Males (*n*=149)**[†]	**Females (*n*=181)** [††]	**Not indicated (*n*=1)**
Baseball	41	—	—
Basketball	8	26	—
Cheerleading	—	4	—
Cross country/track and field	19	40	—
Field hockey	—	16	—
Football	36	—	1
Golf	2	1	—
Gymnastics	—	4	—
Ice Hockey	18	8	—
Lacrosse	6	21	—
Soccer	7	22	—
Softball	—	24	—
Swimming and diving	9	16	—
Tennis	4	—	—
Volleyball	—	3	—

[†]*n*=1 competed in soccer and track and field

[††]*n*=1 competed in soccer and track and field; *n*=1 competed in soccer and softball; *n*=1 competed in field hockey and track and field; *n*=1 competed in track and field and volleyball

Figures

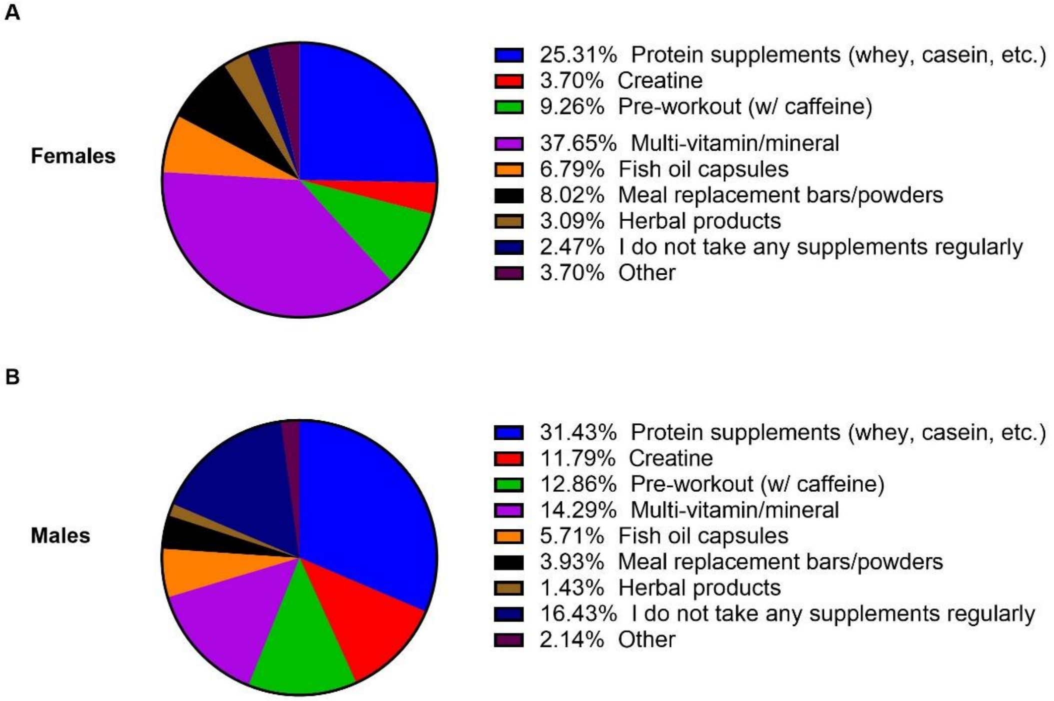

Supplementary Figure S1. Supplements typically consumed by athletes. Athletes were asked to select all supplements they typically consume or if they do not take supplements regularly. Data is presented as percentage of all responses cast among all options (listed in figure). A: *n*=162 total responses; B: *n*=280 total responses.
